# Impact of the COVID-19 pandemic on community-dwelling older adults: A longitudinal qualitative study of participants from the Hertfordshire Cohort Study

**DOI:** 10.1371/journal.pone.0275486

**Published:** 2022-10-14

**Authors:** Ilse Bloom, Jean Zhang, Julia Hammond, Gregorio Bevilacqua, Wendy Lawrence, Kate A. Ward, Cyrus Cooper, Elaine M. Dennison

**Affiliations:** 1 MRC Lifecourse Epidemiology Centre, University of Southampton, Southampton, United Kingdom; 2 NIHR Southampton Biomedical Research Centre, University of Southampton and University Hospital Southampton NHS Foundation Trust, Southampton, United Kingdom; 3 NIHR Musculoskeletal Biomedical Research Unit, University of Oxford, Oxford, United Kingdom; 4 Victoria University of Wellington, Wellington, New Zealand; Oxford University Hospitals NHS Foundation Trust, UNITED KINGDOM

## Abstract

**Background:**

Older adults have been especially vulnerable to adverse effects from the COVID-19 pandemic including higher mortality and more severe disease complications. At the same time, social isolation, malnutrition and physical inactivity are serious concerns among older adults. The pandemic and associated restrictions may serve to exacerbate these issues, presenting increased risks to physical and mental health. The aims of this qualitative study were: i) to explore how community-living older people in the UK experienced the first wave of the COVID-19 pandemic, specifically how it impacted their well-being and associated health behaviours; ii) to explore how older people’s experiences and behaviours changed over time throughout the first wave.

**Methods:**

Qualitative data were collected by conducting serial telephone interviews, with an interval of approximately three months. Participants were from the Hertfordshire Cohort Study, all aged over 80 years. Discussions were audio-recorded, information related to the COVID-19 pandemic was transcribed verbatim and transcripts analysed thematically. Interviews were conducted from March to October 2020.

**Results:**

Data for twelve participants (7 men and 5 women) from a total of 35 interviews were used, comprising two or three timepoints per participant. Analysis identified five overarching themes: 1) shopping strategies and food accessibility, 2) limitations on activities and going out, 3) disruption to healthcare, 4) social and psychological repercussions, and 5) coping strategies. Findings highlight challenges associated with accessing shops, healthcare, and usual activities due to pandemic-related restrictions. Longitudinal findings showed that for some, the ongoing pandemic and related restrictions appeared to aggravate mental health issues (low mood, anxiety) over time, as well as greater feelings of isolation or loneliness, reduced activity and functional limitations; this was despite some relaxation of restrictions later on. Coping strategies used by participants included finding ways to keep busy and to do physical activity safely, maintaining social contact remotely, and having an optimistic or positive outlook, a ‘do what you can’ attitude.

**Conclusions:**

Interventions are likely to be needed in the wake of the COVID-19 pandemic to support health behaviours, such as increasing physical activity, social engagement and improving mental health among community-living older adults.

## Introduction

In March 2020, coronavirus disease 2019 (COVID-19) was declared a pandemic by the World Health Organization (WHO) [1]. Older adults have been especially vulnerable to adverse effects from COVID-19, including higher mortality and more severe disease complications, with greater risk of death and other adverse outcomes of infection in those with comorbidities and frailty [2–4].

Before the advent of the COVID-19 pandemic, malnutrition, physical inactivity and social isolation, were already serious concerns among older adults. Poor diet quality and inadequate micronutrient intakes had been found to be common in older adult populations [5–8], with malnutrition presenting a significant challenge to health in later life [9, 10]. Furthermore, low levels of physical activity were prevalent amongst older adults [11, 12] and social isolation and loneliness were significant issues [13, 14].

Evidence has emerged that COVID-19 pandemic-related restrictions, put in place in many countries including the UK to limit the spread of the virus, may have served to exacerbate some of these issues, at least in the short term. Survey studies have indicated decreased physical activity levels among older adults during the COVID-19 pandemic, particularly during its first wave [15–18]. A number of studies have shown increased feelings of loneliness and greater social isolation in older people surveyed during this initial wave of the pandemic [17, 19, 20]. While some studies suggest changes to dietary intakes in this period, the nature of the impact, particularly among older adults is less clear [17, 21]. Given the important role that health behaviours, such as diet and physical activity, and social relationships play in the promotion of health and prevention of disease among older people, detrimental impacts on these aspects could present increased risks to both physical and mental health, potentially accelerating decline in functional ability and cognitive function, which may be potentially irreversible [22–30].

Some qualitative research has explored the impact of the COVID-19 pandemic on community-living older adults and on their lived experiences during the first wave of the pandemic. The term ’community-living older adults’ broadly encompasses adults aged 65 years and over living in the community; it does not usually consider older adults who are living in residential care or nursing homes. Studies have mostly focused on ages ≥65 years, in various countries including the United States [31, 32], Canada [33, 34], Switzerland [35] and the UK [19, 36]. A large mixed-methods study of adults aged 55 and older (mean (SD) age: 67.3 (7.9) years) in the US collected qualitative data on coping strategies employed by older adults during the early phase of pandemic [32]. One Canadian study specifically investigated the experiences of isolation and loneliness in rural areas, among community-dwelling adults aged ≥65 years (age range: 65 to 89 years) [34]. A study of Scottish older adults, aged 60 years and older (mean (SD) age: 67.3 (5.4) years), used qualitative data to explore the impact of pandemic-related social distancing measures on loneliness, social contact and support, and well-being [19], with qualitative findings reported for participants aged <80 years. Another qualitative study on the experiences of UK older adults, aged ≥70 years (average age 79 years), including = 10 (50%) participants in their 80s or 90s, explored how their well-being and mental health were impacted during the pandemic [36].

Overall, while most qualitative studies have included participants aged ≥80 years, most study populations have tended to be younger on average (usually with a greater proportion of participants in their 60s and 70s), and to our knowledge there have been no longitudinal qualitative studies in this area. Hence, the aims of this study were: i) to explore how community-living older people in the UK, aged over 80 years, experienced the first wave of the COVID-19 pandemic, specifically how it impacted their well-being and associated health behaviours; ii) to explore how older people’s experiences and behaviours changed over time throughout the first wave. The study spanned a phase of the pandemic prior to the start of the UK COVID-19 vaccination programme in December 2020 and to the rollout of the rapid testing programme to the general public in the Spring of 2021.

## Methods

### Participants

Participants were selected from an established cohort of community-dwelling older adults, the Hertfordshire Cohort Study (HCS), which includes individuals born in the county of Hertfordshire, in England, UK, between 1931–1939, and still living there in later life [37]. As part of this larger study, in 1998–2003, 3,225 participants agreed to be interviewed at home. In late 2019 and early 2020, 176 of these participants were recruited into the NAPA (Nutrition and Physical Activity) Study, a randomised controlled trial of an intervention to support behaviour change (Healthy Conversation Skills) [38] aiming to test effectiveness of this approach in improving physical activity and nutrition outcomes in older adults. In this approach a trained researcher or health professional asks open discovery questions (generally beginning with ‘What’ or ‘How’) to help individuals identify something that they would like to change in their lifestyle and supports them to achieve these changes. Following a baseline home visit, participants in the intervention group received follow-up telephone calls, the main aim of these being to assess if participants were on track with the goals set at baseline, and to facilitate goal adjustment if necessary. In the course of undertaking these follow-up telephone calls, participants spoke of how COVID-19 was affecting their daily lives. For this qualitative study, data were included for analysis from NAPA Study participants who fulfilled the following pre-defined criteria: they were in the intervention arm, they had received a telephone call in March 2020, after the start of the official UK national lockdown (23 March 2020), and had received one or two subsequent calls up to October 2020.

### Data collection

Qualitative data were collected in individual interviews conducted over different timepoints, with an interval of approximately three months. Telephone interviews took place between March and October 2020, following a semi-structured discussion guide to facilitate the intervention-related discussion of the main NAPA Study. Discussions evolved organically to include topics related to the COVID-19 pandemic and restrictions, with researchers prompting participants with questions about how they were being impacted, such as, how their physical activity, shopping or eating habits had been affected.

Interviews were conducted by two of the authors; IB, a nutritionist and post-doctoral researcher, and JZ, a medical doctor and PhD student, both trained in qualitative methods and interview skills. Interviews were audio-recorded and information related to the COVID-19 pandemic was transcribed verbatim. The researchers made field notes after each interview, which were compiled into a case report for each participant and were used to facilitate data interpretation. Ethical approval was obtained from the East of England—Cambridgeshire and Hertfordshire Research Ethics Committee, reference number 11/EE/0196. All participants gave written informed consent before taking part in the NAPA Study, including consent to for the telephone calls to be audio-recorded. Consent to audio-record was re-confirmed verbally before each telephone interview commenced.

### Data analysis

Thematic analysis of the data was conducted [39]. The transcripts were read, initial codes were identified to classify the data from all interviews at all timepoints, and these were grouped into themes. Data were coded inductively (data-driven), whereby data were coded into themes and categories without adhering to a pre-conceived framework [39]. A coding frame was created to show these themes, and was used by two researchers (IB and JH) to independently double-code all transcribed data. Coding was completed for each individual (i.e. the unit of analysis) at each timepoint of data collection. The researchers met regularly to discuss the coding, agree the coding frame and to discuss further analysis, including any changes over time.

A longitudinal qualitative study design was used to enable an enhanced understanding of the effects of changes over time [40]. We adopted a trajectory approach to longitudinal qualitative data analysis as described by Grossoehme and Lipstein, which focuses on individuals’ experiences over time [41]. Analyses were conducted after all data were collected. After being coded, the data were organised into matrices, with one matrix per individual. Next, the analysis focused on how the data, arranged into themes, changed or did not change over time; another matrix was used to organize these results. Matrices enabled visual comparisons to be made over time [41, 42]. Comparisons were made between the three time points; each theme was compared over time for all participants, as well as overall change over time for each participant.

## Results

### Participant characteristics

Data for twelve participants (7 men and 5 women) from a total of 35 interviews were used. For all but one of the participants, data were included from three timepoints (for this one participant only 2 timepoints were included as their third interview took place outside of the pre-defined timeframe). Interviews conducted at Time 1 spanned from 23 to 31 March 2020; Time 2 from 18 May to 19 June 2020; and Time 3 from 3 August to 6 October 2020. The descriptive characteristics of the participants are summarised in **Table 1**. All participants were white British. Only one participant had been asked to shield, i.e. they were contacted by the UK National Health Service, having been considered clinically extremely vulnerable, and were formally advised to stay at home for at least 12 weeks from 23 March 2020.

**Table 1:** Characteristics of participants (n = 12); these were assessed in 2019–2020 (prior to the COVID-19 pandemic).

	Median	IQR
Age (years)	83.0	81.5–85.8
Number of comorbidities	2	1–2
Age left education (years)^a^	16.5	16.0–19.5
	**N**	**%**
Social class^a^		
Non-manual	5	41.7
Manual	7	58.3
Living arrangements		
Living alone	7	58.3
Living with a partner	5	41.7
Socially isolated^b^	1	8.3

^a^ Assessed at the HCS baseline (1998–2003)

^b^ Lubben Social Network Scale score <12

### Thematic analysis

Five overarching themes were identified: 1) shopping strategies and food accessibility, 2) limitations on activities and going out, 3) disruption to healthcare, 4) social and psychological repercussions, and 5) coping strategies. These are described below, with any changes over time highlighted and quotations used to illustrate each theme. Changes in each theme over time are also highlighted at the end of this section (in **Table 2**).

**Table 2:** Summary of findings for trends in each theme’s change throughout the first wave of the COVID-19 pandemic for all study participants combined.

	Theme 1: Shopping strategies and food accessibility	Theme 2: Limitations on activities and going out	Theme 3: Disruption to healthcare	Theme 4: Social and psychological repercussions	Theme 5: Coping strategies
**Summary** *Trends in the theme’s change over time for the group*	Time 1: difficulty with doing/obtaining shopping (due to restrictions, lack of availability…). Some participants were receiving help from friends/family	Times 1 and 2: many unable to do their usual physical activity or exercise due to pandemic-related restrictions.	Disruption evident at Times 1 and 2 for some. However, effects of disruption to healthcare for some participants became more evident at Time 3	For some, issues of loneliness/isolation, fear of the virus, impact on mental health (e.g. worry, anxiety), evident at all Time points, but less evident at Time 1.	No evident trend
				Times 2 and 3: relaxation of restrictions lead some to see more friends/family/social groups, despite some restrictions still being in place. Despite further relaxation of restrictions at Time 3, for some, mental health issues (low mood, anxiety) appeared to have aggravated over time, as well as greater feelings of isolation or loneliness	
		Relaxation of restrictions, especially at Time 3, lead some to resume usual activities. For others, this was less the case			
	Time 2: Those who had been receiving help, still getting help.				
	Time 3: Those who had been receiving help, moving towards doing shopping themselves				

#### 1. Shopping strategies and food accessibility

Many participants received help with shopping from family or neighbours during the first wave of the pandemic. This was linked to both positive feelings, for example, sense of appreciation, as well as negative feelings, for example, embarrassment and not always getting exactly what they wanted to eat. Other participants continued to do their own shopping for a variety of reasons, for example, not wanting to put others in danger from exposure to the virus or to be a burden on others. Some preferred to shop regularly to avoid feeling isolated, or due to limited storage facilities.

*‘…I prefer to get my own shopping you know, and I think it’s a burden to leave it to somebody else, but I have had plenty of offers from people if I am stuck … normally I shop everyday because if I don’t go out everyday, I don’t talk to anybody…’ (Participant 8, woman, time 1)*

Particularly at Time 1, there were issues with accessibility of foods, for example, inability to access certain shops (local butchers or market) to buy specific foods and certain foods being less available.

The initial difficulties at Time 1 related to shopping, with some participants getting help from friends or family, appeared to continue at Time 2, with those who had been getting help still doing so.

*‘I got two daughters, so they’re keeping us well supplied [with groceries] …’ (Participant 6, man, time 2)*

At Time 3, those who had been getting help were moving towards doing shopping themselves, and this was perceived as a positive development. **Table 3** displays the experience of one participant, as an example of these events.

**Table 3:** Example case study, with illustrative quotations, of issues related to shopping and food accessibility during the COVID-19 pandemic.

Participant 4 –Woman, living alone	
**Time 1 (March 2020)**	*‘I’ve got at least five people [neighbours] … they’re insistent that I don’t go to the supermarket … they’re doing my shopping for me … I really feel that I mustn’t go against them because if they’re prepared to do it …’*
	*‘I do make my own bread but there’s been no strong flour around*, *so it’s been a problem and eggs were a problem … I use quite a lot of eggs … it’s been a bit ad hoc at the moment … if this is going to be ongoing*, *I need to have a bit more strategy about it*, *a bit more organising*, *so I’m working that out at the moment …’*
**Time 2 (June 2020)**	*‘I mean the thing is with this sort of shutdown thing it’s been much more difficult in some ways*, *you’d think it would be easier … because my neighbours are very good about doing my shopping … whilst I make a list*, *if the thing’s not available*, *I got one lady who’s very good and she’s sort of on the same wavelength and she can give me something else*, *but it’s surprising how much time that takes up in a week actually…’*
**Time 3 (September 2020)**	*‘…I have been out shopping … sometimes you just need to do your own shopping*, *people are very good*, *but it’s all right when you just want milk or you know standard things*, *but some things I’d rather choose for myself…’*

#### 2. Limitations on activities and going out

Many participants were unable to do their usual physical activity or exercise (e.g. cardiac rehabilitation class, walking group) due to pandemic-related restrictions, especially at Times 1 and 2.

*‘I walk quite fast … my gym programme includes a bit of cycling and a bit of walking and then all the all the weights and resistance stuff and I sort of miss that at the moment … I haven’t got the facilities at home basically … I am neglecting the muscle side I have to say and that’s a bit of a concern…’ (Participant 1, man, time 2)*

Particularly at Time 1, there appeared to be differences in how participants interpreted Government guidance on COVID-19 regarding whether they should go out or not, despite not having been advised to shield.

*‘…The roads are nice and quiet and if someone’s coming the other way, I can walk in the road make sure I miss them … I’ve got a river which I can walk along as well…’ (Participant 9, man, time 1)*

*‘No, I don’t go out at all at the moment (Researcher: ‘Were you told by your GP not to go out now?’) No, I haven’t seen my GP, it’s what we were told by the government, I’m 85 now…’ (Participant 12, man, time 1)*

Longitudinal findings indicated that although relaxation of restrictions, especially at Time 3, appeared to lead some participants to resume their usual activities (for example, going to the gym, dancing or walking group), for others, this relaxation did not appear to translate into an increase in going out or going back to their usual activities. To illustrate these developments, the experiences of two participants are reported in **Table 4**.

**Table 4:** Example case studies relating to Theme 2 (limitations on activities and going out), with illustrative quotations.

Participant 7 –Man, living alone	
**Time 1 (March 2020)**	*‘I’ve got to go out on Wednesday ‘cos I’ve got to pick a repeat prescription up … I’ll have to go in the car because it’s quite a way*, *there’s hardly any buses running at all here now …’*
**Time 2 (June 2020)**	*‘Like most of my dancing friends we all want to get back to dancing*, *all absolutely fed up … we had this big thing down in [place] in July we were staying in a hotel for about 4 days*, *this big dinner dance … so that’s been cancelled…’*
	*‘I’ve not been on a bus*, *I mean I’m a bit frightened of getting on the bus because you’ve got to grab hold of those handles and that’s a worry*, *and I find … the only place that [social distancing] happens is outside the supermarket … [elsewhere*, *other people] make no effort to get out of your way or make distance …’*
**Time 3 (September 2020)**	*‘I go sort of once a week the market stalls … and I’ve started dancing again*, *that’s 2 nights a week*, *it comes under adult education and training would you believe*, *there’s only about 8 couples allowed … doing it since the beginning of August…’*
**Participant 10 –Woman, living alone**	
**Time 1 (March 2020)**	*‘…For the last three weeks of course there hasn’t been a great deal of physical exercise I’ve been housebound*, *my age means that I was told to self-isolate from the word go*, *so I haven’t done much exercise*, *I have today and yesterday for the first time gone out for a walk*, *I decided that they said we could take exercise walking … I feel better for it…’*
**Time 2 (June 2020)**	*‘It’s getting a bit easier because I can go out now and walk about a bit more than I was allowed to before*, *but it’s been a long time … all your things you belonged to and went to are curtailed at the time*, *so you can’t get back into those as yet…’*
**Time 3 (August 2020)**	*‘… I still do my walking*, *and I’ve been into town once or twice*, *I find that doesn’t hold any great pleasures for me*, *the town isn’t what it was …’*
	*‘… I find that lots of things like church*, *which was always very regular for me*, *I haven’t been since the beginning of lockdown … I’ve given myself a deadline*, *I think perhaps by the beginning of September I’ll see*, *I’ll try one Sunday and go down…’*

#### 3. Disruption to healthcare

Some participants spoke of cancelled hospital appointments or community prevention groups (e.g. diabetes prevention group), postponed surgeries (e.g. cataracts, hip replacement) or delays in scheduling other medical procedures. For some participants, disruption was evident throughout; for example, even though some cancelled appointments were re-booked, other aspects, such as physiotherapy and diabetes prevention group did not resume.

*‘…Been going to the physio at the hospital but of course that was cancelled because of this virus…’ (Participant 6, man, time 1)*

*‘I’ve had 2 of my 3 hospital appointments caught up because they were cancelled at the beginning of all this lockdown … our meetings have been cancelled you see the diabetic meetings…’ (Participant 5, woman, time 2)*

Disruption to healthcare for a few participants became evident later at Time 3 (for example, a long waiting list for surgery), once their health had already been significantly affected. There was mention of unwillingness to have hospital appointments to avoid being a further burden to the NHS or due to fear of catching or transmitting the virus.

*‘…Because of the long time with NHS I’ve decided to go private [about having a hip replacement]… my mobility is very limited … I’m almost crawling around the house at the moment … the hip’s just been getting worse and what with Covid-19 and all the rest of it …’ (Participant 4, woman, time 3)*

*‘… I have a very sore hip joint … I’ve noticed it [pain] coming on but I’ve managed to keep it under wraps … but in the current situation I can’t obviously take it any further forward because I don’t want to put any further strain on the NHS, I can live with it, but I can’t do what I want to do … with this current situation I’m loath to go into hospitals shall we say too often … I’ve got to think about [wife] as well ‘cos her immune system is shot by the medication she takes so we can’t take any chances or risks, so if it means me suffering a little bit of pain, I’ll suffer a little bit of pain …’ (Participant 9, man, time 3)*

#### 4. Social and psychological repercussions

Participants emphasised the issue of loneliness and isolation, especially those living alone, as well as fear of the virus. Restrictions led to a loss of purpose for some, along with related impact on mental health (e.g. worry, anxiety).

*‘I’m fine, no we’re, a bit depressed obviously under the circumstances, [wife] is shielded…’ (Participant 9, man, time 2)*

*‘Well, tell you the truth I’m fed up to the teeth with the situation … it boils down to the fact that I’m very lonely, I seem to have lost me appetite, I’ve lost weight…’ (Participant 8, woman, time 3)*

*‘…But I think it’s [pandemic] catching up a bit with everybody now, finding it very difficult with um you know life, but I just plod on I live my life one day at a time I’m afraid …’ (Participant 5, woman, time 3)*

For some, these issues appeared to link to physical activity and diet being deprioritised.

*[Participant had been trying to eat more vegetables, and had found some recipe ideas] (Researcher: ‘So you printed some recipes … and did you find some that appeal to you?’) Not at the moment no no, ‘cos all this thing’s come up and uh mind’s elsewhere, at the moment I’m eating steak and kidney pies and all this sort of stuff that I don’t normally eat …’ (Participant 7, man, time 1)*

Longitudinal findings indicated that for some participants, a relaxation of restrictions led to seeing more friends, family and a return to engagement with some social groups, despite some restrictions still being in place; this appeared to be the case for some more than others.

*‘…We’re trying to organise another walk [with walking group] for later this month … a lot of people don’t want to go out, they don’t want to socialise they’re still frightened of what may be a second wave and the virus might come back or whatever, but you know I take the [view] where I’m ever so old I just get on with my life as best I can, given the circumstances you know…’ (Participant 2, man, time 3)*

For some participants, the continued pandemic, with restrictions and fear of the virus, appeared to be linked with aggravated mental health issues, such as low mood and anxiety, as well as greater feelings of isolation or loneliness, and greater difficulty to cope or be motivated, even despite some relaxation of restrictions at Time 3. **Table 5** shows the experiences of two participants, to illustrate these developments.

**Table 5:** Example case studies of deterioration in social and psychological aspects linked to issues related to the COVID-19 pandemic: Illustrative quotations from two participants.

**Participant 3 –Woman, living with partner**	
**Time 1 (March 2020)**	*‘I think one of the main things is for me if I lose my sense of humour … a friend rang me this morning … and she is so funny and she really cheers me up (laughs) which is good…’*
**Time 2 (May 2020)**	*[In the context of discussion about the pandemic and restrictions on usual activities]*
	*‘…All the supermarkets … they’ll only let one person in from each household*, *which I can understand and that’s fine*, *but when you’re old and you’re frightened of dropping something that’s not good*, *it rather knocks your confidence a bit…’*
	*‘I think going out there and doing something that you like is good for your mental health apart from anything else … I was a bit down one day last week and I thought ‘got to snap out of this’*, *but I keep in touch with friends either e-mail or ring them you know so*, *but it would be nice*, *really nice to go out and meet friends … that would help me a lot actually … like we belong to the National Trust and we go to their talks*, *and not being able to physically see them [due to COVID-19 restrictions]*, *that to some extent it’s a bit depressing*, *but then everyone’s in the same boat*, *but that doesn’t help…’*
**Time 3 (August 2020)**	*‘Since lockdown though we’ve managed*, *but I find not meeting the friends that I met for lunch and all this sort of thing and groups*, *I found it quite quite stressful at times…’*
	*‘We have seen the family*, *they were banned for about the first 3 months*, *I think … fortunately the weather was nice so they would stand out in the garden and we’d be inside and we’d have a chat … we have kept in touch with people which is good*, *I think because we’re quite sociable that has been the hardest part of it*, *not meeting…*
	*‘I found with lockdown OK I’ve accepted it because that’s the right thing to do*, *but … I don’t seem to be able to think straight*, *I think there’s this fear I think psychologically at the back of my mind that I don’t want to get this Covid…’*
**Participant 12 –Man, living alone**	
**Time 1 (March 2020)**	*‘… I find at the present time [since the COVID-19 lockdown started] that really now rescheduling your day I find I stay an hour longer in bed because there’s no point in getting up…’*
**Time 2 (June 2020)**	*[In the context of discussion about the pandemic and restrictions on usual activities]*
	*‘It was doing the exercises and that I found that I couldn’t do it*, *perhaps it’s my mental state that wouldn’t fit to doing them you know … it was an extra thing to do*, *which I could do without … at the moment*, *it was just keeping alive was the most important thing you know…’*
	*‘… I walked down through the village for the first time for about 10 weeks and that’s the trouble it [exercises] was just an extra thing for me to worry about you know … up until fairly recently because I’m over 80 I couldn’t go out anyway … well I’m starting to do now … it’s opening up a new*, *a new life really now you can do things…’*
	*‘…Normally on a Tuesday I go to [name of church] and meet up with about a dozen people and we have a chat and we have tea and coffee and that and I do the same in [place] on a Wednesday morning*, *but … haven’t done those for 10 weeks or more … so it’s just meeting different people as well you know … three societies I belong to*, *they’ve all cancelled their [meetings]…’*
**Time 3 (August 2020)**	*[In the context of discussion about the pandemic and restrictions on usual activities]*
	*‘…It’s a question of surviving to stay alive … it’s very difficult being on one’s own … before this virus business I used to have places I could go to in the week … well since this has happened*, *I get confused whether it’s Tuesday or Wednesday … well I’m still trying to do that [walk to village] but see when one did it*, *none of the shops were open*, *it wasn’t worth going down there really … I always went down to the library to read The Times in the afternoon*, *well that’s not open yet*, *so really it’s better to just stay at home you know and survive (laughs)’* *‘…The big problem is being on your own … to do something it’s a mental effort you know…’*
	*‘If you’re on your own*, *like I am now*, *when I put the phone down*, *I’ll just sit here and then I might switch the television on for the news at 6 o’clock or something like that … and now being at my age it’s so much easier to not to do anything…’*

### 5. Coping strategies

Coping strategies used by participants included using a meal delivery service for ready-meals, finding ways to keep busy and to do physical activity safely, maintaining social contact online or by telephone, and having an optimistic or positive outlook, a ‘do what you can’ attitude.

*‘Normally I go to the gym you see, and part of my programme is a fairly fast walk on the treadmill … but of course they’ve stopped so I need to go and do the walking … I went at least twice, three times last week’ (Participant 1, man, time 2)*

*‘…Before the shutdown I bought all the paint necessary to decorate 3 rooms, so I’ve got those really lined up for when perhaps it turns a bit wet … tomorrow we should have been away in our caravan but of course that’s all been cancelled now … but none the poorer for that’ (Participant 11, man, time 1)*

*‘I just seem to have lost enthusiasm … I’m so tired that it is a great effort to actually cook something … I did order some meals from there [meal delivery company] and they were very good, and I ordered some more…’ (Participant 8, woman, time 3)*

There were no obvious changes over time for this theme.

## Discussion

This study explored the experiences of community-living older people in their 80s, living in the English county of Hertfordshire in the UK during the first wave of the COVID-19 pandemic, specifically how it impacted their health and well-being, and associated behaviours. It also investigated how participants’ experiences and behaviours might have changed throughout this wave. Findings highlight considerable challenges associated with accessing shops and healthcare, and limitations on usual activities due to restrictions. Although findings highlight the resilient nature of older adults in terms of their coping and adaptability during COVID-19, longitudinal findings appear to show that for some, the continued pandemic and related restrictions appeared to aggravate mental health issues (low mood, anxiety) over time, as well as greater feelings of isolation or loneliness, even despite some relaxation of restrictions later.

Other qualitative data has highlighted the changes in social activity among older people during the pandemic, and that the experiences of the first wave varied markedly between older adults [19], which is also born out in our study. Some of our findings are consistent with those from another qualitative study of UK older adults, aged over 70 years, that found various threats to well-being during lockdown, such as a fear of hospitalisation and of leaving the house due to the virus, an inability to engage with activities that might support well-being, such as social groups and exercise classes, and a lack of routine [36]. Similar to our findings, the study also identified protective aspects such as more time for new hobbies, the maintenance of a routine and a sense of purpose, and connection with others.

Some of our findings relating to coping strategies are like those from qualitative studies in older adults in the US and Canada, including the adaptation of daily routines and seeking out social engagement and social support [32, 33]. In accordance with our findings, these studies also reported the use of cognitive strategies by older adults, such as the adoption of a positive outlook and positive mindset to deal with the ongoing challenging situation brought about by the pandemic.

Quantitative studies have reported a deterioration of mental health and well-being in older adults as the COVID-19 pandemic unfolded [43, 44], findings which lend support to some of those from this qualitative study. Longitudinal data from the English Longitudinal Study of Ageing [44] showed an increase in the prevalence of depressive symptoms in older adults (mean (SD) age: 67.7 (10.6) years) from before the pandemic (12.5%) to June and July 2020 (22.6%), increasing further at the start of the second wave (28.5% in November and December 2020). There was also an increase in loneliness and an increase in levels of anxiety in this population; the prevalence of anxiety increased from 9.4% in June and July 2020 to 10.9% in November and December 2020).

We found that some participants appeared to hold the incorrect belief that the Government had initially instructed older people, without any underlying health conditions, to not leave their homes at all, which could mean that some people might have restricted their physical activity unnecessarily. This might have had an additional negative impact on people’s functional capacity and mental health. Furthermore, findings also suggest that with the relaxation of pandemic-related restrictions later on in the first wave, while some participants returned to their usual activities, others did not; in part, this could have been to minimise their risk of exposure to the virus, but it is possible that it might also be linked to other factors, such as the potential impact of the pandemic on their mental health. These findings are consistent with some studies that have found a substantial decrease in physical activity level during the COVID-19 pandemic; for example in one study from Belgium, half of the participants (mean (SD) age: 73 (7) years) reported that their activity level had decreased [43]. Moreover, the authors found that an increase in depression was associated with a decrease in physical activity level during the pandemic [43]. Notably, some participants in our study appeared to be experiencing worsening mobility due to musculoskeletal health issues, and this might have further contributed to functional limitations and reduced activity. Additional research is needed to investigate further how mobility and participation in physical activity might have changed over time among older adults during the pandemic, including beyond its initial wave; indeed one such study is currently under way in Canadian community-living older adults [45].

### Strengths and limitations

While much of the research on the effects of the pandemic on older adults and their perspectives has included participants aged 80 years and over to some extent, most studies have primarily focused on slightly younger ages, whereas the present study provides insight into the experiences of older people specifically aged 80 or older and how they have been affected by the pandemic and associated restrictions. Although this was a small sample, the longitudinal nature of the qualitative data provided 35 data collection points, which has uniquely enabled an examination of how older people’s experiences and behaviours changed over time during the initial months of the pandemic. This has provided clues as to the factors that might contribute to potentially long-lasting effects on mental and physical health and well-being.

Due to the pragmatic nature of this study, in terms of its pre-defined inclusion criteria (in order to focus on the first wave of the pandemic), and that coding was carried out after data collection, we were not able to monitor data saturation. As such, data saturation was not necessarily reached, and we cannot rule out that, if further interviews had been held, additional topics or themes relevant to the research question might have emerged. In addition, given that the focus of the main study in which this qualitative study was embedded was nutrition and physical activity (the NAPA study), the focus of the interview discussions did primarily centre on these topics; therefore, some of the findings from the present study reflect the emphasis on this subject matter.

Whilst this study includes participants with a range of living arrangements and levels of socio-economic status, it should be highlighted that the study sample was not designed to be representative of the wider UK older population. All study participants were white British older adults, so it is not possible to draw any conclusions about other aspects that may have relevance for other ethnic groups. This study focused only on the first wave of the pandemic and further research is needed to explore how subsequent waves, along with developments such as the roll-out of the UK vaccination programme in late 2020 and of widespread rapid testing, impacted on older adults’ behaviours and well-being.

## Conclusion

The findings from this study suggest that some UK older adults have experienced a range of changes during the first wave of the COVID-19 pandemic. Despite the myriad coping mechanisms adopted by study participants to deal with challenging circumstances, findings suggest that some might have faced prolonged issues throughout the first wave, such as low mood and anxiety, feelings of loneliness and isolation, reduced activity levels and functional limitations.

Interventions are likely to be needed in the wake of the COVID-19 pandemic to support older adults to improve health behaviours, reduce loneliness and social isolation, and support mental health and access to healthcare. Local authorities and healthcare professionals who encounter older people should usefully support older adults to identify positive coping strategies. Awareness should be raised among older people and their carers of the importance of maintaining healthy diets and physical activity levels, as well as meaningful social relationships, with particular attention given to support older adults who might still be fearful of attending certain settings, including crowded spaces. There is a need for early screening by healthcare professionals to detect issues as they arise, including a review of mental wellbeing, and provision of appropriate advice or signposting to relevant services. The reluctance of some older people to attend hospital or GP appointments due to a fear of the virus, suggests that there might be a need for healthcare professionals to proactively contact such older patients. In the event of a future crisis, similar to that of the COVID-19 pandemic, policymakers both at local and national levels, should put in place relevant interventions, such as awareness raising campaigns on the issues highlighted above, as well as implementing or ensuring ease of access to measures such at-home exercise initiatives, phone or online befriending and grocery/meal delivery services to those who might benefit from them, to help mitigate the negative impact of the pandemic on this particularly vulnerable age group. Interventions could be designed to adapt and adhere to the requirements of any future pandemic-related measures, such as social distancing.

Whilst the focus of the analysis has been about the COVID-19 pandemic, findings from this study could inform our understanding of how community-living older adults could be supported to increase their resilience in the face of a variety of changing circumstances that are likely to impact their health and well-being, for example, retirement, bereavement, and medical events. Further studies are necessary to explore the unintended longer-term impacts of COVID-19 pandemic-related measures on health behaviours, social isolation and loneliness, and associated health outcomes, and the reversibility of changes experienced during the pandemic.

## Supporting information

S1 File COREQ checklist. (PDF)
